# A Review of Single-Cell RNA-Seq Annotation, Integration, and Cell–Cell Communication

**DOI:** 10.3390/cells12151970

**Published:** 2023-07-30

**Authors:** Changde Cheng, Wenan Chen, Hongjian Jin, Xiang Chen

**Affiliations:** 1 Department of Computational Biology, St. Jude Children’s Research Hospital, Memphis, TN 38105, USA; changde.cheng@stjude.org; 2 Center for Applied Bioinformatics, St. Jude Children’s Research Hospital, Memphis, TN 38105, USA; wenan.chen@stjude.org (W.C.); hongjian.jin@stjude.org (H.J.)

**Keywords:** scRNA-seq analysis method, cell-type annotation, single-cell data integration, cell–cell communication

## Abstract

Single-cell RNA sequencing (scRNA-seq) has emerged as a powerful tool for investigating cellular biology at an unprecedented resolution, enabling the characterization of cellular heterogeneity, identification of rare but significant cell types, and exploration of cell–cell communications and interactions. Its broad applications span both basic and clinical research domains. In this comprehensive review, we survey the current landscape of scRNA-seq analysis methods and tools, focusing on count modeling, cell-type annotation, data integration, including spatial transcriptomics, and the inference of cell–cell communication. We review the challenges encountered in scRNA-seq analysis, including issues of sparsity or low expression, reliability of cell annotation, and assumptions in data integration, and discuss the potential impact of suboptimal clustering and differential expression analysis tools on downstream analyses, particularly in identifying cell subpopulations. Finally, we discuss recent advancements and future directions for enhancing scRNA-seq analysis. Specifically, we highlight the development of novel tools for annotating single-cell data, integrating and interpreting multimodal datasets covering transcriptomics, epigenomics, and proteomics, and inferring cellular communication networks. By elucidating the latest progress and innovation, we provide a comprehensive overview of the rapidly advancing field of scRNA-seq analysis.

## 1. Introduction

Single-cell RNA sequencing (scRNA-seq) has revolutionized the fields of biology and medicine by enabling exploration of the transcriptomic profiles of individual cells, work that has opened a new window onto the heterogeneity of cells and the communication networks that exist among them. This scRNA-seq has proven invaluable in the identification of rare malignant cells, aiding in the study of cancer biology and personalized medicine [1,2].

In combination with advancements in spatial transcriptomics ([3,4,5,6], and additional methods outlined in Table 1), as well as single-cell genomics, epigenomics, and proteomics, scRNA-seq has emerged as a powerful and versatile tool for both basic and clinical research. It has particularly advanced knowledge in fields like immunology and oncology [7,8,9], which require a deep understanding of cellular dynamics and interactions in order to develop effective therapeutic strategies and improve patient outcomes [10,11].

To effectively analyze transcriptomic data at the single-cell level, numerous pipelines and methods have been developed to tackle the significant challenges posed by technical variability, low cell abundance, and the presence of diverse cell types. Once the initial data processing is complete, a wide range of downstream analyses can be performed. These include cell annotation, where cells are categorized into distinct cell types and cellular states, and the integration of findings across patients, datasets, and different modalities. Additionally, inferring cell–cell communication based on the expression of genes encoding ligands and receptors is another important aspect of scRNA-seq data analysis.

Despite significant advancements in scRNA-seq analysis, certain challenges persist that require further investigation. One such challenge is the process of clustering, which plays a critical role in characterizing cellular heterogeneity, identifying rare cell types, and analyzing cell–cell interactions. Existing cell clustering methods often struggle to accurately determine the number of cell types or clusters, resulting in uncertainty in the resolution estimation. Consequently, the manual classification of cells heavily relies on domain expertise.

A recent benchmarking study [33] conducted a comparative analysis of various approaches to inferring the number of cell types. These approaches were categorized into inter-class vs. intra-class similarity methods, such as scLCA [34], and community-detection-based methods, exemplified by Monocle3 [35,36,37]. The findings of this study revealed that community-detection-based methods generally performed favorably, whereas inter-class vs. intra-class similarity methods produced significant variability. It should be noted, however, that high clustering performance does not guarantee an accurate estimation of cell types [33].

Additionally, current clustering algorithms often fail to incorporate the intrinsic hierarchical structure among cells, leading to potential inaccuracies. However, the recent introduction of RNA-seq clustering techniques that integrate biological realities into their models [38] holds promise for the future development of multi-level, multi-scale clustering strategies that are tailored specifically to scRNA-seq analyses.

It must be acknowledged that there are theoretical constraints (such as those suggested by Kleinberg’s Impossibility Theorem), which may prevent the development of an optimal clustering solution that satisfies a predetermined set of reasonable properties [39]. As a result, it may be worthwhile to consider an algorithm that generates a series of clusters at different scales and contexts [40].

Clustering algorithms that consider the hierarchical structure of cells not only enhances the effectiveness of scRNA-seq analysis but also offers a further direction for research. This review concentrates on several challenges, such as obtaining and modeling count data, cell annotation, multimodal integration, and cell–cell communication. It is organized as follows.

Section 2 explores in detail statistical modeling approaches for UMI-based and non-UMI-based scRNA-seq count data. Understanding the intricacies of count data and employing appropriate statistical models are critical for the accurate analysis and interpretation of scRNA-seq results.

Section 3 covers cell-type annotation and clustering, vital steps in scRNA-seq data analysis. Defining a cell type is not a trivial question [41]. Properly and efficiently annotating cells involves employing various computational tools. These tools can be categorized into unsupervised and supervised cell annotation methods. Key components of the cell annotation workflow include known marker gene databases, signature scoring, well-annotated reference datasets, and supervised modeling techniques. Obtaining biologically meaningful cell types, subtypes, and supertypes is challenging yet essential for a comprehensive understanding of scRNA-seq data.

Section 4 explores data integration strategies. Integrating scRNA-seq data with other modalities is critical for gaining a holistic understanding of complex biological systems. Spatial barcoding techniques, for instance, facilitate the mapping of annotations derived from scRNA-seq data and the precise localization of cell types. Integrating scRNA-seq data with other single-cell modalities such as single-cell ATAC-seq, ChIP-seq, or protein data provides insight into regulatory networks and signaling pathways. Additionally, integrating multiple scRNA-seq datasets makes it possible to identify both common and rare cell types, compare gene expression patterns under different conditions, and construct comprehensive reference atlases. Such integration enhances the power of scRNA-seq analysis and facilitates the exploration of cellular heterogeneity across tissues and organisms. To integrate diverse omics data measured at the single-cell level, various methods including mapping, deconvolution, and multimodality fusion are employed.

Section 5 covers the inference of cell–cell communication. The scRNA-seq provides a powerful tool for studying intercellular communication, offering a deeper understanding of ligand–receptor signaling and intercellular interactions. The single-cell resolution of scRNA-seq allows for the identification of rare cell populations and variations in ligand and receptor expression, enabling the characterization of different cell types within a population. By investigating gene expression across different time points or developmental stages, researchers can identify changes in ligand–receptor interactions and signaling cascades, shedding light on the dynamics of intercellular communication over time. Integrating scRNA-seq data with other omics data, such as proteomics or spatial transcriptomics, further enhances the interpretation and functional relevance of the identified communication networks. Overall, scRNA-seq is a powerful approach for unraveling cell–cell communication, enabling the identification of ligand–receptor interactions, deciphering cellular heterogeneity, and providing valuable insights into the mechanisms underlying various biological processes, development, tissue homeostasis, disease, and therapeutic interventions.

We conclude the review by highlighting the advancements and efforts made to tackle the technical challenges of data annotation, data integration, and cell–cell communication inference in scRNA-seq analysis. 

## 2. Statistical Count Modeling for scRNA-Seq and Spatial Transcriptomics

**Figure 1:**
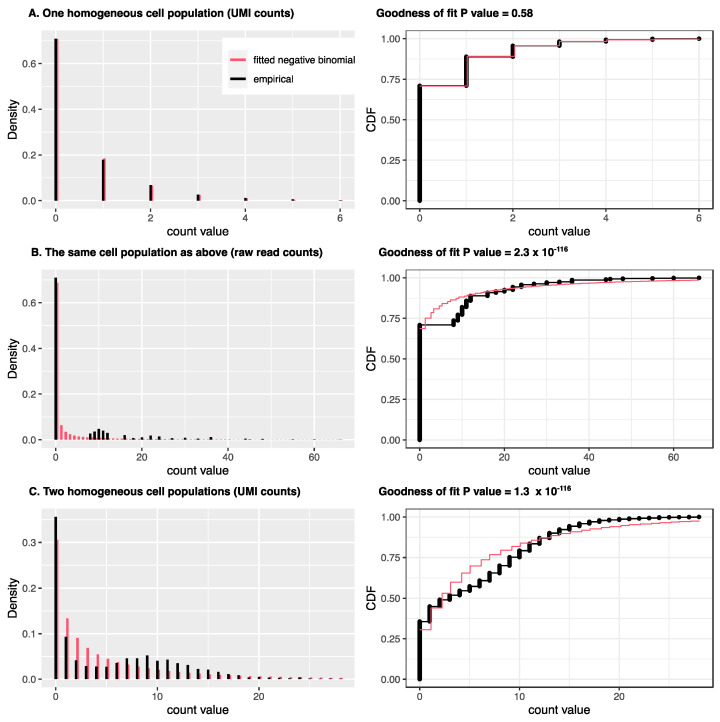
Distinct statistical models for unique molecular identifier (UMI) counts and raw read counts. For homogeneous cell populations, UMI counts can be effectively modeled using the negative binomial distribution. In panel (**A**), counts are simulated using a negative binomial distribution. Raw read counts are derived in panel (**B**) by multiplying UMI counts from panel (**A**) by a factor between eight and 12, introducing the dropout phenomena. In the presence of heterogeneous cell populations (panel (**C**)), a single negative binomial distribution may not accurately capture the complexity. Instead, a negative-binomial-based regression model incorporating covariates representing different cell types can be employed. The left panel displays density plots, while the right panel illustrates cumulative distribution functions (CDFs).

Depending on the technology used, different models should be employed for data analysis. Due to low capture efficiency, for most expressed genes, scRNA-seq data may only capture zero or one copy of the mRNA from the raw material [42]. This observation is less obvious if the raw read counts are used because PCR amplification in scRNA-seq protocols can convert one mRNA copy into over ten or more copies, leading to the so-called drop-out phenomenon [43]. However, unique molecular identifier (UMI) technology can label the original mRNA molecules before PCR amplification and convert multiple copies, due to amplification, back into a single copy. Chen et al. [44] showed the first direct evidence that statistical modeling should be different depending on whether UMI counts or raw read counts are used. Specifically, as illustrated in Figure 1, when UMI counts are used, there is no need to use zero-inflated models, which were widely used for scRNA-seq data modeling in the early stages of scRNA-seq platforms with raw read counts. Later investigations reached similar conclusions [45,46,47,48]. UMI counts can be modeled either using a negative binomial (NB) distribution or a Poisson distribution with the mean further modeled with other distributions, such as Gamma (equivalent to NB) or log-normal distributions. 

This distinct modeling is also reflected in methods integrating different single-cell data sets, especially those modeling the raw count matrix directly. For example, the deconvolution method Cell2location [49], Stereoscope [50], and DestVI [51] all use NB distribution to model scRNA-seq data sets. The mapping method gimVI [52] uses either NB distribution or zero-inflated NB (ZINB) distribution depending on the data sets; scANVI [53] uses the larger model ZINB to accommodate data sets from different platforms. 

For spatial transcriptomics, a recent study [54] evaluating statistical count modeling, including both the high-plex RNA imaging (HPRI) and spatial barcoding techniques, suggests that zero inflation is not necessary. It shows that the excess zeros are more likely due to cell heterogenicity, such as different cell types. Since the resolution of HPRI is at the single-cell level, and no PCR amplification is used for in situ methods, it is not surprising that the count modeling is similar to that for modeling UMI counts in scRNA-seq data sets. For example, the gene expression imputation method gimVI uses Poisson distribution for smFISH and NB distribution for starMAP in its count modeling. In spatial barcoding techniques, such as 10x Visium, high-definition spatial transcriptomics (HDST), and Slide-seq/Slide-seq2, there are multiple cells in a spot instead of a single cell. However, for these UMI based techniques, negative binomial or Poisson-lognormal models seem to work well for each spot when the models can account for different proportions of cell types within spots [49,51].

## 3. Cell-Type Annotation

Multicellular organisms consist of cells that can be categorized by their function and morphology. Single-cell transcriptomics makes it possible to individually profile thousands of cells in multiple tissues and organisms within a single experiment. The computational workflow of single-cell RNA sequencing (scRNA-seq) comprises several crucial steps.

First, quality control removes outlier or low-quality cells and genes. Then, cells are clustered and visualized in a two-dimensional map using techniques like t-distributed stochastic neighbor embedding (tSNE, [55]) or uniform manifold approximation and projection (UMAP, [56]). The subsequent essential steps include clustering and cell-type annotation. Clustering involves grouping cells with similar gene expression patterns into distinct clusters. Determining and labeling cell types or states on the map is known as cell-type annotation or identification. This process aids in understanding cellular heterogeneity and facilitates downstream analyses such as cell–cell interactions and data integration.

Recognizing the hierarchical structure of cell types is crucial for understanding cell function and interactions. However, the taxonomy or hierarchy is not often carefully considered during cell annotation.

In recent years, a variety of methods and computational tools have been developed to identify cell types, broadly categorized as unsupervised or supervised approaches. Proper and efficient annotation of cells or clusters into biologically meaningful types, subtypes, and supertypes is a non-trivial task. Key components in the annotation workflow include signature databases, scoring methods, well-annotated reference datasets, and supervised modeling.

### 3.1. Cell Annotation by Signature Scoring

The prevailing method of cell-type annotation consists of unsupervised clustering analysis followed by manual or automatic annotation using a set of known “marker genes”, also known as gene sets, markers, or signatures. An example of this approach is the Seurat function FindMarkers [57], which employs differential expression analysis to identify biomarkers defining clusters. This annotation approach does not necessitate training a model with another “annotated” reference dataset. Still, it heavily relies on existing biological knowledge of known marker genes and involves subjective decision-making, such as choosing the number of clusters (resolution).

Moreover, this process is typically manual, leading to potential time constraints and annotation inconsistency.

#### 3.1.1. Signature Database

Several databases provide extensive collections of known markers that can aid in cell-type annotation (see Table 2). These databases include MSigDB [58], Enrichr ARCHS4 tissues [59], TISSUES 2.0 [60], SaVanT [61], xCell [62], celldex [63], PanglaoDB [64], CellMarker [65,66], SCsig, and CellMatch [67]. Among these, PanglaoDB, CellMarker, SCsig, and CellMatch were specifically developed for scRNA-seq analysis. The scMRMA method utilizes Cell Ontology [68] to reorganize PanglaoDB into a hierarchical structure, enabling consistent representation of cell types across various levels of anatomical granularity.

#### 3.1.2. Scoring Method

Common scoring methods, like single sample gene set enrichment analysis (ssGSEA, [69]), gene set variation analysis (GSVA, [70]), and Singscore [71], were initially designed for bulk RNA-seq data. The ssGSEA score quantifies the coordinated up- or down-regulation of an input gene signature within a sample. GSVA performs kernel density estimation of the gene expression profile across all samples, and Singscore calculates a normalized mean percentile rank. However, these methods rely on statistical assumptions that do not consider the extensive presence of zero values and missing genes within individual cells across a dataset, making these bulk-sample-based methods prone to dropout effects and therefore suboptimal for scRNA-seq data analysis.

The optimal scenario for scoring genes is when there is a bi-modal distribution, indicating a high expression of signature genes in one cell type but not others. However, at the single-cell level, most genes are either not expressed or exhibit unstable expression patterns. Gene expression analysis is further complicated by dropouts (resulting from low input of RNA amounts), transcriptional stochasticity, and diversity of cell states and identities.

Researchers have made significant efforts to address these challenges in order to improve the evaluation of gene signatures in scRNA-seq data. Several approaches have been developed, including the cell-type activity (CTA) score [64], single cell signature scorer (SCSS, [72]), ModuleScore (implemented in Seurat’s AddModuleScore function), AUCell [73], Ucell [74], JASMINE [75], scType [76], scCATCH [67], and scMRMA [77], among others (see Table 3). These methods aim to provide improved assessments of gene signatures within scRNA-seq datasets.

The cell-type activity (CTA) method calculates an activity score for each cell type by summing the weighted expressions of its marker genes [64]. The SCSS score for a signature in a cell is computed as the sum of all UMI (unique molecular identifier) counts for the genes in the gene set expressed in that cell divided by the sum of total UMI counts in the cell.

Seurat’s AddModuleScore function calculates the average expression levels of each signature at the single-cell level, with the aggregated expression of control feature sets subtracted. The analyzed features are grouped into bins based on their average expression, and control features are randomly selected from each bin.

AUCell utilizes the area under the curve (AUC) to determine whether a critical subset of genes in the input gene set is enriched at the top of the ranking for each cell. The AUC reflects the proportion of expressed genes in the signature and their expression values relative to other genes within the cell.

UCell calculates gene signature scores for scRNA-seq data using the Mann–Whitney U statistic, which is correlated with the AUC scores computed by AUCell. JASMINE calculates the approximate mean using gene ranks among expressed genes and measures the enrichment of the signature in expressed genes. These two values are scaled to a range of 0–1 and averaged to obtain the final JASMINE score.

ScType calculates a cell-type-specific marker enrichment score per cluster by computing a cell type specificity score for each marker, and then multiplying these by the z-score of marker gene expression across all cells. The values of each cell signature are summed across cells corresponding to a specific cluster, resulting in the cluster summary enrichment score.

scCATCH employs the evidence-based scoring (ES) process, which utilizes tissue-specific cell taxonomy reference databases (CellMatch) to determine cell types and subtypes in two steps.

Notably, scMRMA utilizes the CTA scoring method with different parameters at different levels (major cell types and subtypes). This approach enables multiresolution cell annotation through iterative clustering and the mapping of clusters to the hierarchical PanglaoDB marker database.

By implementing scoring methods, the annotation process of cells or clusters can be efficiently automated in annotation tools like scType, scCATCH, and scMRMA. Since single-resolution unsupervised clustering cannot capture both global and local biological variances simultaneously, a multi-resolution strategy like scMRMA can achieve more comprehensive and detailed annotation.

The performance of signature-based cell annotation relies on several factors, including gene sets, scoring methods, and the characteristics of the query data. It is important to note that the signature scores obtained may not always be normalized or comparable across different gene sets or datasets. Improving the reproducibility and reliability of cell annotation will require addressing the following general limitations:

1. Cell marker databases are compiled from diverse data sources generated using different technologies, each with its own technical biases such as sensitivity, dropouts, and cell population purity. The derived signatures for the same cell type can therefore vary across technologies. Additionally, signatures obtained from bulk RNA-seq or microarray data may not accurately annotate cell types in single-cell data.
2. There is a lack of consistent criteria or methods for curating signatures. Gene sets can be derived experimentally, computationally, or manually curated from the literature. Even computational selection methods, such as differential expression analysis, can result in different gene sets due to arbitrary cutoffs (e.g., log2 fold change, false discovery rate, top number of genes).
3. The size of gene sets (i.e., the number of genes they contain) varies greatly, making it difficult to compare the scores of different signatures. Smaller gene sets (e.g., size < 20) are more likely to yield cells with unstable scores, while larger gene sets (e.g., size > 100) can provide greater stability for detection and evaluation. It is often observed that the signature scores of large random gene sets follow an approximately normal distribution, abiding by the central limit theorem.
4. Redundancy across gene sets is common in large databases. Since gene sets may share a significant proportion of their constituent genes, scoring results can be dominated by long lists of candidate cell types associated with overlapping signatures, potentially obscuring meaningful cell types that possess only a few marker genes.
5. Most databases adopt a flat structure, treating each cell type equally and independently. While this approach can effectively distinguish major cell types, it may struggle to identify cell subtypes due to the lack of relationships between cell types. Hierarchical cell type databases could enhance discrimination of specific cell types or subtypes [77].
6. Unstandardized cell nomenclature in certain publications can lead to overlapping or ambiguous anatomy terms or identifiers for cell types. To address this, collaborative efforts such as the Cell Ontology (CL) and The Human Cell Atlas (HCA) have begun to build a high-dimensional compendium of cell information.

For quality control of signature-based annotation, the following measures can be considered:

1. Assess the reliability of cell annotation by plotting the score histogram of a specific gene set and examining the distribution of scores within cell types in the dataset.
2. Visualize the signature scores or average expression of a gene set in a two-dimensional plot. Calculating the mean expression with library-size normalization provides an intuitive approach.
3. Some methods are sensitive to the number of detected genes or dropout rates. Checking marker gene expression through dot plots or stacked violin plots can help to identify potential issues.
4. Employ a confusion matrix or mosaic plot to evaluate the final assignment of cell type labels.

By addressing these considerations and implementing quality control measures, the reliability and reproducibility of cell annotation based on signatures can be improved.

### 3.2. Cell Annotation by Supervised Learning

In recent years, supervised cell annotation has gained significant attention due to the exponential growth of publicly available single-cell RNA sequencing (scRNA-seq) data, including projects like the Human Cell Atlas (https://www.humancellatlas.org/ accessed on 25 May 2023, [78]), Tabula Muris (https://tabula-muris.ds.czbiohub.org/ accessed on 25 May 2023, [79]), and the Mouse Cell Atlas (https://bis.zju.edu.cn/MCA/ accessed on 25 May 2023 [80]). Supervised learning, a type of machine learning, has been employed to transfer cell type labels from labeled to unlabeled datasets for cell-type annotation. Various common algorithms, such as Support Vector Machine (SVM, [81]), Random Forest [82], k-nearest neighbors (kNN, [83]), neural networks [84], and deep learning [85], have been utilized in this field.

In general, the process of supervised cell annotation involves several steps. Firstly, a classifier is constructed using a reference dataset of known cell types, which serves as the labeled training set. Secondly, feature selection is performed to identify the most informative features for training the classifier. Thirdly, the classifier is trained using the labeled training set to associate specific features with each cell type. Finally, once the classifier has been trained and evaluated for its accuracy, it can be used to predict the cell type of new cells or clusters in an unannotated dataset.

As these steps require substantial computational expertise, numerous automatic annotation software tools employing different supervised approaches have been actively developed to enable efficient supervised cell annotation.

#### 3.2.1. Feature Selection

Feature selection is a crucial step in enhancing the performance and interpretability of a model by identifying the most informative variables within a dataset. The primary objective is to reduce the dimensionality of the feature space by eliminating redundant, irrelevant, or noisy features. This reduction not only improves computational efficiency during model training and evaluation but also facilitates more accurate machine learning outcomes.

When it comes to cell-type annotation, known marker genes associated with specific cell types, obtained from external resources, can be directly employed as features. Alternatively, marker genes can be identified through differential expression (DE) analysis, which involves comparing the gene expression levels in a particular cell type against all other cell types using statistical tests like t-tests [86], Wilcoxon signed-rank tests [87], or dedicated packages such as limma [88], DESeq2 [89], or Seurat’s FindAllMarkers function.

Certain feature selection methods rely on variance filtering. By establishing a threshold on the variance, features below that threshold are eliminated from the feature set. Bartlett’s test [90] is utilized to assess whether the variances across all groups are equal. Additionally, F-statistics are useful if the data follows a normal distribution and the group variances are equal. Several feature ranking methods, such as information gain (Entropy test) [91], chi-square statistics [92], the Kolmogorov–Smirnov (KS) test [93], and the bimodality index [94], can assign scores, ranks, or significance levels to genes based on their relevance to cell-type annotation. Genes with higher scores or significance levels are considered more informative or cell-type specific.

Li et al. [91] pioneered the use of entropy, a measure of dispersion from information theory, to assess the distribution of gene expression levels following a Poisson–Gamma mixture model. The entropy could be estimated directly from the logarithm of the mean gene expression, and genes with larger total entropy differences were found to be more cell-type specific. FEAST [95] applies unsupervised consensus clustering followed by an F-test on the clusters to calculate feature significance and rank features accordingly. Andrews et al. [96] introduced M3Drop, which employs a Bayesian model to estimate the dropout rate for each gene, incorporating its mean expression, and subsequently performing differential expression analysis to select informative genes. This dropout-based feature selection method demonstrates superior performance compared to variance-based approaches. Lin et al. [97] showed that the differential expression (DE) gene selection method outperformed other tested methods (DE, DD, BD, and DP) in terms of cell-type annotation accuracy (Table 4).

#### 3.2.2. Prediction Model (Classifier)

A variety of methods have been developed to annotate cell types in single-cell transcriptomics data using machine learning models. For instance, scPred [98] employs support vector machine (SVM)-based classifiers on PCA-transformed gene expression matrices. The singleCellNet [99] and scAnnotate [100] methods utilize the Random Forest technique for classification. Garnett [101] trains a multinomial classifier using elastic-net regression [102] to discriminate between different cell types. The L2-regularized logistic regression implemented in cellTypist [103] enables automated annotation of immune cells across human tissues. The scClassify [97] method takes advantage of a k-nearest neighbors (kNN)-based learning algorithm, combining multiple similarity metrics and feature selections. On the other hand, scDeepSort [104] employs a weighted graph neural network, while Cell Blast [105] leverages large-scale reference databases and an autoencoder-based generative model to obtain low-dimensional representations of cells and employs a cell similarity metric for mapping query cells to specific types. SciBET [91] achieves rapid and accurate single-cell-type identification using a multinomial-distribution model and maximum likelihood estimation. Notably, scBERT [106] is an adaptation of the Bidirectional Encoder Representations from Transformers (BERT, [107]) model, originally developed for natural language processing for cell-type annotation. The scBERT method incorporates gene expression data to represent cells and their relationships, demonstrating superior performance in tasks such as novel cell type discovery and robustness, to batch effects, through to pretraining and fine-tuning.

Several supervised cell annotation methods have been specifically developed for single-cell RNA sequencing (scRNA-seq) data (Table 5), focusing on the correlation between the target and reference datasets. Notable methods include SingleR [63], CellAssign [108], CHETAH [109], and scmap [110]. SingleR assigns cellular identities to single-cell transcriptomes by comparing them to a built-in reference transcriptome of pure cell types obtained from microarray or bulk RNA-sequencing data. CellAssign employs a probabilistic model that utilizes a marker-based reference for cell type assignment. CHETAH adopts a hierarchical classification approach, allowing cells to be assigned to intermediate or unassigned types through stepwise traversal of the classification tree. Finally, scmap classifies query cells based on their similarity to reference cell types using various correlation measures.

Supervised methods are generally not optimized for discovering novel cell types. Without additional configurations to prevent over-classification, any new cell type in the target data may be forced into one of the existing cell types in the reference dataset. However, a common strategy is to set a threshold on the prediction odds, classifying certain cells as unassigned. This threshold-based approach is implemented in popular tools such as scmap, CellAssign, and CHETAH, allowing the identification of unassigned cells.

The assessment of prediction results can be effectively conducted using multiple established metrics, each providing a unique perspective:

1. Accuracy: This metric captures the ratio of correctly classified cell types to the total number of cells, providing a broad view of model performance.
2. Adjusted Rand Index (ARI): ARI allows for the comparison of clustering patterns between the predicted and actual (ground truth) classifications. It offers an insight into how closely the model’s clustering aligns with the actual data.
3. F1 score: The F1 score offers a robust measure of a model’s classification accuracy. It amalgamates precision and recall into a single measure by averaging the individual F1 scores for each class. It provides a more nuanced view of model performance, especially in scenarios where class imbalances exist.
4. Normalized Mutual Information (NMI): NMI is a metric that quantifies the shared information between the predicted and ground truth distributions. By normalizing against the maximum possible mutual information value, it gives a relative perspective on how much the predicted labels reveal about the actual labels, which is particularly useful in clustering contexts.
5. Variation of Information (VI): VI evaluates the degree of difference between predicted and actual labels. It effectively gauges how much the model’s classification deviates from the true label distribution.

There are more metrics that have been used to evaluate the performance of cell clustering and annotation; interested readers may consult Hossin et al. [112].

The performance of cell annotation methods is heavily influenced by the quality of annotated reference databases. However, constructing these reference datasets presents several notable challenges. One of these challenges is the unavoidable need for manual cell-type annotation, which can be a time-consuming and subjective process. Additionally, determining the appropriate clustering resolution or the number of cell types in both the reference and query data often relies on subjective choices based on specific study requirements or expert opinions. Another crucial factor affecting classifier accuracy is the quality of the training set. If the reference data is not well curated, the classifier may yield inaccurate results, leading to erroneous cell-type annotations in the query data. These considerations underscore the importance of meticulous curation and careful selection of reference datasets for robust and reliable cell-type annotation.

### 3.3. Other Cell Annotation Methods

#### 3.3.1. Cell-Integration-Based Label Transfer

An alternative method for annotating cells based on transcriptomic data involves integrating a query dataset with a well-established reference dataset using an integration algorithm. This integration enables the annotation of clusters that span both datasets, allowing the transfer of labels from the reference data to the corresponding query cells within the clusters. This approach facilitates the identification of identical, distinct, and novel cell types. However, it is important to note that this method can be computationally demanding. Additionally, integration algorithms may exhibit varying performances, and batch effects or disparities between the reference and query data can introduce challenges. Further discussion of these aspects, including single-cell data integration, will be presented in Section 4.

#### 3.3.2. Semi-Supervised Annotation

Semi-supervised learning [113,114,115] is a machine learning approach that leverages both labeled and unlabeled data during model training. This technique is particularly valuable when only a limited amount of labeled data is available, as the unlabeled data can enhance the model’s understanding of the problem domain. By incorporating unlabeled data, the model can learn more about the underlying patterns and structure of the data, leading to better generalization. This approach is particularly useful when acquiring labeled data is costly or time consuming, as it can make the most of available resources and achieve satisfactory results with a smaller labeled dataset. However, it is important to note that training a semi-supervised model can be computationally intensive [114,116]. Additionally, selecting the appropriate algorithm for a given problem and interpreting the results of such a model can be challenging.

There are two noteworthy recent implementations in this field: SCINA [117] and scNym [118]. SCINA is a semi-supervised model that utilizes an expectation-maximization algorithm [119] to annotate cells at the cluster level. It achieves this by fitting a bimodal distribution to cell type marker genes. On the other hand, scNym is a semi-supervised approach that employs an adversarial neural network [120] to transfer cell identity annotations from one experiment to another. Remarkably, scNym has demonstrated high performance in cell-type annotation across experiments, even when faced with biological and technical differences.

In summary, semi-supervised learning is a valuable technique that can enhance the performance of machine learning models when labeled data is limited. Recent implementations such as SCINA and scNym showcase the potential of semi-supervised approaches in annotating cells at the cluster level and transferring annotations across experiments.

### 3.4. Perspective

In many tissues, there are typically a small number of major cell types [121]. These major cell types can further be divided into subtypes in a hierarchical manner, forming what is known as a “cell type hierarchy” [122]. While most supervised methods classify cells directly into a “terminal” cell type, this one-step annotation approach can successfully identify the major cell types but may result in misclassification of similar cell subtypes.

To address this challenge of cell subtyping, and considering the hierarchical relationships between cell types, recent advancements in scientific research have introduced multi-scale or multi-resolution classification frameworks such as scMRMA and scClassify. These frameworks take into account the hierarchical relationships between cell types and aim to improve the accuracy of cell subtyping. Additionally, the divisive hierarchical clustering method uses various marker genes to cluster cells in multiple iterations and at different resolutions, as seen in the co-occurrence clustering algorithm [123] and TooManyCells [124].

Interestingly, a similar approach based on multi-level scale-adaptive clustering has been reported for the unsupervised classification of tumor subtypes using RNA-seq. This approach, known as Resolution-Adaptive Coarse-to-Fine Clusters Optimization (RACCOON, [38]), classified more than 13,000 samples into an eight-level hierarchical tree based on their expression similarities. It successfully generated an atlas consisting of 455 tumor and normal classes. Building upon this extensive hierarchy, the same research group developed a classifier called OTTER for childhood cancer. OTTER is an ensemble of convolutional neural networks that performs robustly across all cancer types.

The choice of cluster resolution in data analysis depends on the specific dataset and research objectives. Low-resolution clustering can impede the accurate identification of distinct cell types, while annotating cells at the single-cell level is susceptible to errors due to stochastic noise. To overcome these challenges, several approaches have been proposed.

A common strategy is to employ validation indices, such as the silhouette score or the gap statistic. These indices evaluate clustering quality by comparing the distances within clusters to those between clusters. A higher score indicates better clustering performance. An example of this approach is scLCA [34], which combines the Tracy–Widom test [125,126,127] based on random matrix theory to determine the number of significant eigenvalues, and the silhouette score to rank the results of spectral clustering. The scLCA approach has demonstrated effectiveness in accurately determining the number of clusters in scRNA-seq data through systematic benchmarking [33].

Another approach involves utilizing visualization tools like t-SNE or UMAP. These techniques aid in identifying clusters that may be excessively small or large, assisting in the refinement of cluster resolution. Optimizing resolution in this manner can yield biologically meaningful and desirable outcomes, especially when considering common dropout events in scRNA-seq data.

Nevertheless, it is important to recognize that, while there are various strategies for optimization and hierarchy, the ultimate decision on cluster resolution remains a subjective judgment that the researcher must make.

Nonetheless, the careful curation, integration, and optimization of hierarchical knowledge databases derived from cell-type ontologies and expression similarities in atlas datasets will have a pivotal impact on the advancement of cell-type annotation methodologies. Moreover, this process will enable us to delve deeper into our comprehension of cell heterogeneity in developmental processes and diseases, ultimately facilitating the development of more effective treatments.

The annotation of new or rare cell types or subtypes presents challenges due to the scarcity of known markers or reference datasets associated with them. In such cases, a combination of approaches can be considered. Initially, a supervised method can be employed to predict the major cell types using a well-established reference dataset. Subsequently, an unsupervised clustering method can be applied to identify subtypes within each major cell type separately. When annotating new or rare cell types, a conservative approach is recommended. It is preferable to omit a cell type lacking solid validation rather than erroneously categorizing a cell as a different type.

## 4. Single Cell Data Integration

Integrating scRNA-seq data with spatial transcriptomics and other modalities is essential for understanding complex biological systems [128]. Integrating multiple scRNA-seq data sets can help to identify both common and rare cell types, compare gene expression patterns under different conditions, or build a large reference atlas (see Section 3). Integrating scRNA-seq data with spatial transcriptomics, such as those from high-plex RNA imaging (HPRI) or spatial barcoding techniques, allows for mapping annotations derived from scRNA-seq data, spatial localization of cell types, and deconvolution of spatial-barcoding-based data sets. Integrating scRNA-seq data with other single-cell modalities, such as single-cell genomics, epigenomics, and proteomics, allows for a deeper understanding of the regulatory networks and signaling pathways in cellular processes [129,130]. Simultaneously, measurement of different omics data modalities at the single-cell level enables the application of multimodality fusion methods, which provide a more comprehensive picture of the underlying cell states. We classify single-cell data integration methods into three main categories: mapping, deconvolution, and multimodality fusion, although the boundaries between these categories may not always be clear. We summarize representative methods of each category in Table 6.

### 4.1. Mapping

Mapping methods in single-cell data integration aim to establish connections between entities in different datasets or modalities, or to transform and correct raw datasets so that they can be compared effectively. One common approach is to project the datasets into a shared space of lower dimensions, allowing for meaningful comparisons and addressing irrelevant batch effects. Principal component analysis (PCA) or singular value decomposition (SVD) are frequently employed in popular tools like Seurat V3 [131], Scanorama [132], Harmony [133], and fastMNN [134]. Canonical correlation analysis (CCA), as implemented in Seurat V3, is also a viable option. SpaGE [135] employs a similar technique to CCA for projecting datasets into a common space. Non-Negative Matrix Factorization (NMF) is extended to integrative NMF in LIGER [136], enabling the identification of a shared space between two datasets. In contrast, methods like scVI [137] and scANVI utilize probabilistic models to represent the raw gene count matrix and neural networks in order to embed single cells into a lower dimensional space. Variational Inference (VI) is employed to optimize the model parameters.

Batch effects or platform effects correction is commonly used in the mapping methods. For example, MNN/fastMNN, Scanorama, and Seurat V3 all use mutual nearest neighbors (MNN) to identify matching pairs of cells from different data sets, and then batch correction vectors can be defined as the difference between the pairs. To apply batch correction to all cells in a data set, a weighted average of batch correction vectors for each cell is calculated based on a defined distance between the cell and other paired cells. Harmony iteratively performs batch correction using a linear mixture model and the soft k-means clustering, which adds a penalty term to maximize the independence between the cell cluster label and the batch source. LIGER separates batch effects and biological effects through the shared matrix in integrative NMF, which represents the shared gene-level features. Then, LIGER builds a shared factor neighborhood graph for later graph-based clustering. Seurat V3, Harmony, and LIGER are all designed to integrate not only among scRNA-seq data sets but also between scRNA-seq data sets and other data modalities (Table 1). For scVI and scANVI, the batch IDs are encoded and used as input to the neural network.

There are also methods of learning the mapping directly. For example, Tangram [138] uses a full probabilistic model to model the scRNA-seq data and the spatial data without dimension reduction in order to estimate a mapping between single cells from scRNA-seq and voxels, representing either spatial spots from spatial barcoding or HPRI. One unique feature of Tangram is that it is designed for integrating scRNA-seq with either spatial barcoding or HPRI platforms. The gimVI method, designed for imputing missing genes in HPRI data sets, assumes a shared low-dimensional representation between scRNA-seq and HPRI data and uses a generative probabilistic model to jointly model both modalities.

Where both unimodal data sets, such as scRNA-seq, and multimodal data sets, such as 10x Multiome and CITE-seq, are available, methods have also been developed to integrate them. Both Cobolt [139] and MultiVI [140] use variational autoencoding to project modality data into a latent space and then merge them into a single space. Both single-modal and multimodal data can be integrated in this way. Beyond low-dimensional representation, MultiVI can also be used to generate batch-corrected feature values after integration. Recently, Seurat V5 [141] has applied the dictionary learning method to improve multimodal integration. It first applies mapping methods within the same modality between unimodal and multimodal data sets, and then uses a dictionary representation (weighted linear combination) to represent each unimodal data set; therefore, all datasets are represented in the same feature space. It also utilizes Laplacian eigen-decomposition and data sketching techniques to improve computational scalability. The advantage of multimodal-based mapping is that there is no need to force a common feature unit between different modalities, e.g., there is no need to sum scATAC-seq peaks around a gene to match the gene expression in scRNA-seq.

### 4.2. Deconvolution

Deconvolution methods in single-cell data integration have a clear objective: to estimate the composition of cell types in multi-cell-based data or encoded spots from spatial barcoding techniques (Table 1), given a pre-defined list of cell types. Before the widespread use of single-cell technology, deconvolution methods were already developed for dissecting bulk RNA-seq [142], some of which have been adapted for the single-cell context, such as SpatialDWLS [143].

Most deconvolution methods model the gene expression of mixed cells as a linear combination of expression levels from individual cells. When directly modeling the raw count matrix, negative binomial distributions (used in Cell2location and stereoscope) or hierarchical models based on Poisson distributions (as in RCTD [144]) are often employed. The mean parameter is used to model the linear combination. Additionally, because each gene or spot may deviate from the model differently, these methods also include gene-specific and spot-specific effects in the mean parameters (e.g., Cell2location, stereoscope, RCTD).

SpatialDWLS operates on normalized spatial data and uses an enrichment analysis as an initial step to narrow down the list of candidate cell types. This step only requires a list of marker genes for each cell type. Subsequently, it utilizes dampened weighted least squares (DWLS), originally developed for bulk RNA-seq [145], to analyze spatial data using gene signatures from the candidate cell types.

SPOTlight [146] uses a seeded NMF (non-negative matrix factorization) approach. The initial values of the decomposed matrix are filled with cell types and 1—P values from corresponding DE (differential expression) analysis. As a result, the matrix decomposition of the scRNA-seq matrix represents the feature patterns of cell types and cell type membership. The non-negative least squares (NNLS) method is then applied to decompose the spatial data using the feature patterns from scRNA-seq, allowing estimation of the proportion of different cell types.

Deconvolution methods require cell type information from scRNA-seq data, which can be generated through clustering and assigning cell types or annotation methods as described in the Section 3. The gene signature representing each cell type can be either derived using statistical modeling or by calculating the average of normalized gene expression in the scRNA-seq dataset. It is important to note that mapping methods do not necessitate cell type information from scRNA-seq.

### 4.3. Multimodality Fusion

Differently from mapping, which integrates different data sets, multimodality fusion deals with one data set where at least two different modalities are measured simultaneously. The objective is to integrate information from all modalities to provide a more comprehensive description of cell states, leading to improved accuracy and granularity in cell-type classification or annotation.

MOFA+ [147] is an extension of MOFA [148], employing a statistical framework known as multi-omics factor analysis (MOFA). It decomposes the joint multimodality feature-sample matrix into a product of two low-dimensional matrices, resulting in a simplified representation. This decomposition effectively captures both the shared factors that are common across different modalities and distinct factors that are unique to each modality.

TotalVI [149] utilizes a probabilistic variational autoencoder (VAE) model to extract the low-dimensional cell representation from both modalities measured in CITE-seq.

Seurat V4 [150] adopts a weighted similarity method to combine the similarities computed based on each modality. This approach assigns weights to each modality for every cell, considering their predictive performance on other modalities using the nearest neighbors derived specifically from that modality. This flexible approach seems to work well for different modality fusion, such as CITE-seq, SHARE-seq, and ASAP-seq.

Once the low-dimensional representation or the similarity matrix of cells is available, visualization and clustering methods can be applied based on these embeddings. Both TotalVI and Seurat V4 not only offer low-dimensional representation but also provide scRNA-seq mapping using multi-omics-based references, further enhancing the integration and interpretation of data from different modalities.

### 4.4. Linear and Non-Linear Modeling

There are two classes of dimension reduction techniques in single-cell data integration: linear and non-linear. Linear transformation includes PCA/SVD, CCA, factor analysis, and non-negative matrix factorization. These methods often incorporate non-linear batch correction to address batch effects, such as the MNN method used in MNN/fastMNN, Scanorama, and Seurat V3.

Non-linear dimension reduction methods have also been proposed, including those methods used in scVI, scANVI, TotalVI, and other scVI tools [151]. The scVI and scANVI tools adopt probabilistic methods that directly model the raw counts of scRNA-seq data. A notable advantage of modeling raw counts is the elimination of the normalization step. To introduce non-linearity into the modeling, neural networks are employed instead of linear models to establish the connection between parameters (e.g., the mean gene expression) and the low-dimensional representation. This utilization of neural networks greatly enhances the flexibility of the modeling process. Efficient computation is achieved using variational inference, an approximation method in Bayesian inference.

By incorporating batch information directly into the model, scVI, scANVI, and TotalVI enable the direct comparison of cell states from different experiments and datasets based on the inference of low-dimensional embeddings. This integration of batch information enhances the ability to compare and analyze cells across distinct data sets.

### 4.5. Batch Correction for Cell-Level Analysis and Gene-Level Analysis

Batch correction plays a key role in integrating multiple scRNA-seq data sets. When dealing with data generated from different batches but from the same platform, it is essential to consider both cell-level analysis of the low-dimensional representation of cells and gene-level analysis, examining individual gene expressions.

Several mapping methods, including scVI, scANVI, MNN/fastMNN, Scanorama, and Seurat V3, offer both batch-corrected cell representation and batch-corrected gene expression. These methods allow for comprehensive analysis at both the cellular and gene expression levels. However, methods like Harmony and LIGER provide only batch-corrected cell representation, lacking the capability to directly visualize batch-corrected gene expressions.

These differences among methods may impact the choice of which approach to employ when integrating single-cell datasets. For example, if the visualization of batch-corrected gene expressions is of particular interest, it is crucial to select methods that can provide these corrected expressions as output. Careful consideration of the specific analytical goals will guide the selection of the most appropriate method for integrating and analyzing single-cell data.

### 4.6. Available Benchmark Results

The rapid advancements and widespread adoption of single-cell technologies and spatial transcriptomics have led to the development of numerous methods for single-cell data integration. To provide guidance and recommendations for different application scenarios, it is crucial to conduct benchmarking studies to assess the performance of available methods.

In an early evaluation of batch effect correction methods for scRNA-seq data [152], Harmony, LIGER, and Seurat V3 were recommended. However, this evaluation did not include several recent methods. In a more recent study by Leucken et al. [153], 68 preprocessing-and-method combinations were evaluated on various single-cell data sets for atlas-level, single-cell integration. The results indicated that scANVI, Scanorama, scVI, and scGen [154] were among the top-performing methods overall.

Li et al. [155] conducted a benchmarking study using paired data sets consisting of spatial transcriptomics and scRNA-seq data. They evaluated 16 integration methods for two specific tasks: gene expression imputation in spatial transcriptomics using scRNA-seq data and cell type deconvolution. The results highlight Tangram, gimVI, and SpaGE as the top methods for the imputation task, while Cell2location, SpaitalDWLS, and RCTD performed well for cell-type deconvolution.

These benchmarking results provide valuable insights for users in selecting appropriate methods for their specific needs. As more single-cell datasets are generated, we anticipate the availability of additional independent benchmarking results, further aiding researchers in making informed choices for single-cell data integration.

### 4.7. Assumptions, Potential Limitations, and Future Direction

One main assumption of data integration methods is that there is correspondence between data sets and that at least a subset of cells represents shared biological states, e.g., shared cell types among different data sets. Integrating data sets without tight correspondence can lead to misleading mapping results, such as combining different cell types or cells from diverged species like humans and mice. Most developed methods have demonstrated their performance in the presence of good correspondence. However, it would be valuable to investigate their performance in scenarios where little correspondence exists. Such evaluations can aid in diagnosing potential sample swapping or mismatches, or in testing the hypothesis regarding the existence of correspondence between two samples. For instance, there have been several studies showing the good correspondence of cell types between human and mouse for various tissues ([156,157,158]); however, the similarity in expression profiles of the same cell type is much higher within species than between species [156].

Another assumption made in mapping between different modalities is that there is a straightforward relationship between gene expression measurement and other modalities, such as chromatin accessibility or DNA methylation. However, this assumption may not hold true for developing or transitioning systems. For example, Hao et al. [141] show that gene expression changes can “lag” behind variation in chromatin accessibility, so different modalities may not align simply at the same time or at the same cell development stage.

Spatial barcoding techniques are commonly used for data analysis in molecular biology research. However, one important aspect that is often overlooked is the diffusion of mRNA around the spots during tissue permeabilization. Recent studies have demonstrated the benefits of incorporating a probabilistic model to account for mRNA diffusion, leading to improved analysis results [159]. In addition, integrating information extracted from hematoxylin and eosin (H&E) staining images, which are captured before permeabilization, with the spatial barcoding data set can offer a more robust approach against the effects of mRNA diffusion [160].

Regarding batch effects, a common assumption is that they are largely independent of biology variations, as assumed in MNN/fastMNN and reflected in Harmony’s objective function. Under this assumption, there is no ambiguity between batch effects and biology variations. However, when this assumption is violated, i.e., when batch effects correlate with biology variations, different batch correction methods may yield divergent results based on their individual assumptions. As observed in Leucken et al. [153], different methods exhibit trade-offs between batch effect correction and preservation of biologically relevant information. For instance, when each scRNA-seq sample corresponds to a batch, the batch effects and the relevant group labels might be correlated. Chen et al. [161] evaluated many batch correction methods under this setting for DE (differential expression) analysis and found that MNN/fastMNN, a method recommended from Tran et al.’s evaluation ([152]), can lead to an inflated false discovery rate (FDR) in DE analysis. Extending evaluations in this setting to other important integration methods, including scVI and Seurat V3, would be worthwhile.

In the context of deconvolution for spatial barcoding datasets, an intriguing question arises as to whether deconvolution methods are still necessary when the spot resolution reaches the single-cellular or sub-cellular level. In such cases, while most spots may contain a single cell, a small portion of spots may still harbor doublets or triplets, similar to droplet-based scRNA-seq platforms. Consequently, doublet or triplet detection and deconvolution methods may still be required if the focus is on understanding the composition of cell types within these spots. However, given that most spots represent single cells, using a separate scRNA-seq reference panel for deconvolution may not be necessary. This can help to reduce costs and ensure consistency between the reference and the doublets or triplets.

## 5. Cell–Cell Communication

The transition from single-cell organisms to multi-cell organisms is a significant innovation in organismal architecture that expands the potential for evolution into new life forms [162]. Establishing efficient mechanisms for cell–cell communication to coordinate activities among different cell types for proper high-level function in one individual is thought to be a crucial step in the development of complex multicellularity [163]. Cell–cell communication affects multiple biological processes in a multicellular organism, such as development [164], tissue homeostasis [165], and immune responses [166]. Dysregulation of cell–cell communication is associated with diseases like cancer [167,168] and aging [169]. A comprehensive understanding of intercellular communication can be instrumental in the reprogramming of cellular signaling pathways, leading to the regulation of pivotal biological processes. This knowledge can potentially pave the way for the advancement of cell-based therapies [170]. One of the most important forms of inter-cell communication is the ligand–receptor interaction [171]. A ligand can be a protein that is released by one cell, which binds to a protein receptor on the surface of another cell. Structurally, the transmembrane receptor is significantly more complex than the ligand and comprises three distinct domains: (1) the extracellular ligand-binding domain, (2) the hydrophobic domain that spans the cell membrane, and (3) the intracellular domain responsible for transmitting signals. The ligand–receptor binding event initiates a signaling cascade that invokes a specific cellular response and is characterized by a high degree of specificity. It is noteworthy that each receptor has the capacity to recognize only certain ligands or a group of closely related ones. This specificity is essential for the precise transmission of signals between cells, thereby facilitating the coordination of complex physiological processes. It is interesting how even a small change in certain key residues that affect the receptor’s 3D structure and can significantly alter how a ligand binds to it [172,173]. Although interactions through ligands and receptors are typically studied by quantifying protein abundance, gene expression analysis offers a more straightforward and more accessible way to investigate this communication by measuring the expression of corresponding genes as indirect evidence of protein interactions [174]. However, traditional expression analysis using bulk RNA sequencing may not accurately capture the cellular heterogeneity and complexity of cell interactions. Single-cell RNAseq based cell–cell communication analysis (Table 7), integrated with spatial transcriptomics and multi-omics, addresses some of these limitations by providing a higher resolution and more comprehensive data.

### 5.1. Cell–Cell Communication: Genes’ View

Most cell–cell communication inference using scRNA-seq data can be conceptualized as a form of co-expression analysis, wherein the expression pattern of a ligand from one cell and a paired receptor from another cell are used to infer cell–cell communication mediated by the binding of the respective proteins [174,213,214,215]. However, it is important to note that transcript abundance does not always directly correlate with translated protein abundance [216,217], and the mere expression of ligand–receptor pairs does not guarantee functional cell–cell communication [171,189]. Nevertheless, studying the expression patterns of ligand–receptor pairs using scRNA-seq data can provide valuable insights for data exploration, hypothesis generation, experimental design, and ultimately it can enhance the interpretation and utilization of ligand–receptor expression pattern information in the context of cell–cell communication. In this section, we discuss methods utilizing scRNA-seq data to characterize the expression patterns of ligand–receptor pairs while acknowledging the caveats and limitations.

Before studying ligand–receptor expression patterns, cell–cell communication analysis usually requires grouping cells into types. The uncertainty associated with identifying the cell type or cellular state can significantly impact the subsequent analysis of cell–cell communication. On the other hand, classifying cells into clusters and exploring the communication within these clusters may disregard the heterogeneity of communication at the individual cell level. A comprehensive approach is required to investigate cell communication, considering both the individual cell level and the cluster level. A recent exceptional study uses a binning approach to analyze cell–cell communication at a single-cell resolution [201].

The exploration of ligand–receptor expression patterns begins with quantifying transcript abundance from scRNA-seq data. Various computational approaches have been developed to identify and quantify the expression levels of ligands and receptors in individual cells or cell populations (Table 7). Several well-annotated databases have been developed, beginning with the pioneering work of Ramilowski et al. [218], who systematically compiled a ligand–receptor interaction map from the literature. These databases serve as valuable resources for studying cell-to-cell communication and are often accompanied by published methods for inferring such interactions [175,177,181,182,187,219,220,221]. They encompass a wide range of ligand–receptor interactions, ranging from several hundred to a few thousands [189,222], and are predominantly focused on human and mouse systems (with a couple of exceptions, such as FlyPhoneDB for *Drosohpila* [223] and PlatnPhoneDB for plants [224].) An interesting observation is that there appears to be a greater degree of similarity among databases in regards to their collection of ligands or receptors than their interactions with one another [189]. In addition to ligand–receptor information, some databases also integrate additional regulatory pathway data, further enhancing their utility (i.e., [221]).

Upon determining the expression levels of ligands and receptors, comprehensive statistical and computational analyses can be employed to characterize their expression patterns across diverse cell types, facilitating the inference of cell–cell communication. The interaction or the communication between cells, facilitated by a specific ligand–receptor pair, can be mathematically represented as a function of the ligand’s expression in one cell and the corresponding receptor’s expression in the other. While the precise form of the function utilized to model communication capacity must be determined empirically, it is reasonable to expect that it should exhibit specific properties to effectively measure the potential of cell–cell communication mediated by the expression of the ligand–receptor pair.

In terms of these properties, effective communication necessitates activating both the ligand and receptor, with changes in the ratio of occupied receptors being sensitive to variations in ligand concentrations. Moreover, activating a ligand–receptor channel is anticipated to transmit complex information, suggesting that a scalar may not be sufficient to quantify the extent of cell–cell communication mediated by ligand–receptor interactions. Consequently, multiple metrics may be necessary to characterize cell–cell communication thoroughly, and an ensemble approach may be helpful to efficiently extract information for the cell–cell communication inference [189]. Nonetheless, it is worth noting that the product of the expression levels of an active ligand and an active receptor holds biological significance, as shown by a correlation between expression products and tumor phenotypes in one paper [225]. This is commonly employed in current methods for inferring cell–cell communication, as reviewed and discussed in other works [174,189,213,215,226,227,228,229].

In published studies, a diverse array of functions have been utilized to quantify cell–cell communication, as highlighted in a comprehensive review by Peng et al. [215] However, at a fundamental level, the quantification of cell–cell communication capacity typically follows a general form, with the product of expression levels in ligand–receptor pairs serving as a key component. Specifically, the relationship can be expressed as simply as:

$$s\left(L,R\right)\propto \phi \left(L\right)\times \phi \left(R\right).$$

Here, s(L,R) represents a cell–cell communication score function, while ϕ denotes an activation function. The activation function ϕ captures the influence of ligand (L) and receptor (R) expression levels on the overall communication score. Although the specific form of activation function ϕ may vary across methods, this general formulation offers a flexible framework for assessing cell–cell communication capacity in ligand–receptor interactions.

A commonly employed approach for quantifying the intensity of cell–cell communication involves the utilization of expression thresholding for a given ligand–receptor pair. This methodology generates a class of binary communication score function through a classification process, as summarized in reference [174]. By establishing a suitable threshold, often determined through differential gene expression analysis, the expression levels of a ligand and a receptor can be converted into binary values using step functions:

$$\phi \left(x\right)=\left\{\begin{array}{c} 0\;\;\;\;\mathrm{if}\;x\leq x_{o}\\ 1\;\;\;\;\mathrm{if}\;x>x_{o} \end{array}\right..$$

In this context, when the expression level (x) is above the threshold $\left(x_{o}\right)$, the gene is considered active and assigned a binary value of one. Conversely, if the expression level falls below the threshold, the gene is considered inactive and assigned a binary value of zero. The binary expression values of a ligand and a receptor are then multiplied for the communication score function:

s(L,R)∝L×R.

The resulting binary communication score function possesses the property that cell–cell communication is deemed active $\left(s(L,R)=1\right)$ if and only if both ligand and receptor are active. This property ensures that the communication score accurately reflects the activation status of the ligand–receptor pair.

An alternative and frequently employed strategy for scoring cell–cell communication involves directly taking the product of the continuous expression values of a ligand and a receptor, as described in reference [174]. In other words, an identity activation function is applied before the product operation:

$$\phi \left(x\right)=x.$$

The utilization of the continuous product carries additional information that may be biologically relevant, in contrast to the binary product. However, it is important to note that the continuous product method is not a simple extension of the binary product method and can yield qualitatively different results. The communication score quantified by the continuous product method may be misleading when the product is dominated by a highly expressed ligand or receptor, while its partner exhibits low expression. To illustrate this point, consider the following example involving two sets of ligand–receptor expressions: $\left(L,R\right)=(1,\;1)$ and $\left(L^{\prime },R^{\prime }\right)=(100,\;0.1)$. The continuous communication score method yields a greater magnitude for $\left(L^{\prime },R^{\prime }\right)$ as ${(S}^{\prime }=L^{\prime }\times R^{\prime }=10)$, while for (L,R), (S=L×R=1). However, if we adopt a threshold of one, considering anything larger or equal to one as active, then the binary communication score method results in a greater communication strength for (L,R) as $\left(S=L\times R=1\right)$, compared to ${(S}^{\prime }=L^{\prime }\times R^{\prime }=0)$. It is evident that the choice of communication scoring function can significantly impact the perceived strength of cell–cell communication, highlighting the importance of carefully selecting the appropriate methodology based on the specific context and biological considerations.

An interesting variant of score function is implemented in scSeqComm [200]. This approach involves quantifying the activation function of a ligand or receptor by measuring the probability of observed expression being higher than expected. To simulate a ‘fuzzy logical AND’ operator, their communication score function takes the form of the minimum between the ligand and receptor. By adopting this approach, it minimizes the influence of interacting genes with significantly different expression levels, ensuring that the resulting score is not biased towards a single gene dominating the entire interaction signal.

To enhance the accuracy of modeling cellular communication via ligand–receptor pairs, exploring alternative activation functions or different combinations of functions and evaluating their performance in cell–cell communication analysis may prove useful. For example, the ReLU (rectified linear unit) activation function,

$$\phi \left(x\right)=\max\left(0,x-x_{o}\right),$$

which is commonly utilized in deep neural networks, could be a promising option for this purpose. By incorporating the ReLU function, it is possible to model channel activation while preserving the expression details for activated ligands or receptors.

Other types of activation functions are theoretically plausible for scoring cell–cell communication using scRNA-seq data. One notable consideration is based on the Hill–Langmuir equation [230,231], which has been extensively employed to model ligand–receptor binding and response at equilibrium. The Hill–Langmuir equation takes the form as:

$$\theta =\frac{{\left[L\right]}^{n}}{K_{d}+{\left[L\right]}^{n}}.$$

In this equation, θ represents the fraction of the receptor protein bound by the ligand, n represents the Hill coefficient, while $K_{d}$ represents the equilibrium dissociation constant of the ligand–receptor complex. Therefore, it seems reasonable to consider an activation function defined as follows:

$$\phi \left(x\right)=\frac{x^{n}}{K_{d}+x^{n}}.$$

This activation function, derived from the Hill–Langmuir equation, exhibits a characteristic sigmoidal binding activity. The binding activity of this activation function exhibits a gradual saturation as the concentration of ligands increases. Consequently, at high ligand concentrations, the ability of cells to communicate with each other becomes impractical, as the receptor fails to respond to changes in ligand concentration. This phenomenon has significant implications for interpreting the communication score function, which is a product of various activation functions.

The Hill–Langmuir equation suggests that the communication score function serves as an indicator of the extent of ligand–receptor binding and, in turn, the amount of information transmitted. However, it is important to note that a high communication score may not always signify efficient communication. Instead, it could indicate saturation of the communication channel. Consequently, our understanding of the communication score function may need to consider the possibility of saturation or the relative composition of a cell population.

In summary, the Hill–Langmuir equation-inspired activation function suggests a non-linear nature of ligand–receptor interactions. This model-based approach may allow for better characterization and interpretation of cell–cell communication dynamics within complex biological systems. Cellchat utilized the law of mass action and Hill function to model interactions between ligands and receptors involved in signaling but, interestingly, their equation includes the expression of ligand and receptor as a regularized product [177], similar to what has been done in another work [181].

### 5.2. Cell–Cell Communication: Cells’ View

To characterize the communication state between cells and to identify the pattern of cell-to-cell communication in a dataset, it is necessary to integrate the information gathered from individual ligand–receptor pairs. One way to summarize the ligand–receptor interactions between cells is to count the active ligand–receptor pairs. The total number of active ligand–receptor interactions may serve as an indicator of the overall intensity of communication between cells. This methodology has been effectively employed by Ramilowski et al. [218] in constructing their cell-to-cell communication network. By leveraging the count of active ligand–receptor pairs, the authors could capture and represent the complex landscape of intercellular communication, elucidating the underlying network of signaling interactions. The intercellular network approach greatly facilitates the visualization, exploration, and extraction of high-level information for hypothesis generating in cell–cell communication analysis, which has been adopted by many computational tools [175,177,178,181,182,187,192,221] and numerous analyses (i.e., [179,232,233,234,235,236,237,238]), with popular analysis including hub detection, differentiation tests, or context-dependent variation in intercellular communication networks, with dedicated methods developed for comparing ligand–receptor interaction across environments [204,212]. For example, by using cell-connectivity networks, NATMI could recover the cell type with dominating communicating edges and demonstrate its ability to identify the cell-connectivity change associated with aging through a differentiation network analysis [187]; while in the analysis [232], uterine decidual cells were identified as a cell–cell interaction hub by examining the number of edges in a cell–cell communication network, and the authors carefully interpreted the edges as the cell–cell signaling potentials. By applying results from network theory, it is possible to use centrality and betweenness metrics to identify major signaling sources, targets, mediators, and influencers [177].

An alternative approach to characterizing cellular-level cell–cell communication from sets of ligand–receptor interactions is tensor decomposition and factorization elegantly introduced by two works [193,195]. By representing the interactions between ligands and receptors across different cell types as a tensor or a three-dimensional array, we can effectively capture the complexity of these interactions. The ligand–receptor tensor can then be decomposed into a combination of simple tensors or cell–cell interactions using a generalized process, extending matrix decomposition methods like principal component analysis and singular value decomposition. In essence, the tensor approach is a dimension reduction method, allowing us to summarize and combine individual ligand–receptor interactions into a comprehensive “super” cell–cell interaction. Alternatively, we can begin with a matrix where one dimension represents ligand–receptor pairs and the other represents sender–receiver cell pairs, and then apply established matrix decomposition techniques. A non-negative factorization approach has also been applied to identify key latent communication patterns among signaling pathways [177]. Modularity in cell–cell communication and ligand–receptor interaction, as noticed in one work [218], is probably the biological basis for the decomposition method to work. A new technique has been created that utilizes receptor–ligand modularity to accurately determine the cell–cell communication [211].

Network and pathway approaches provide valuable insights into the impact of ligand–receptor interactions on the cellular state of receiver cells through targeted downstream gene expression, while also enabling the prioritization of candidate interactions between cells or across contexts [177,178,181,186,190,199,212,221]. By leveraging the known activity of target genes associated with ligand–receptor pairs in receiver cells, it is possible to quantitatively assess the intensity of cellular communication using a generative modeling framework [186]. This approach allows us to assign probabilities to communication events. Furthermore, integrating gene expression data from target genes and considering the significance of ligands within known ligand-signaling networks can effectively prioritize active ligand–receptor interactions [178].

On the other hand, a recent analysis shows that the state of receptors, as an entry point of a signal pathway, may not strongly correlate with the state of target genes [239]. Therefore, the approach of integrating the expression pattern of downstream genes may not be as helpful for the pathways in which the states of receptors do not correspond well with target downstream genes. The response time of cellular signals exhibits a wide range, spanning from milliseconds to hours [240]. Consequently, the potential for delayed responses may impact the correlation between the status of the ligand and the corresponding cellular state, as observed in the downstream expression of genes. The extent to which these changes in downstream target gene expression are captured depends on the experimental design and may or may not be deemed relevant.

### 5.3. Cell–Cell Communication: Spatial View

The way that cells communicate with each other depends on where they are located and how far apart they are. There are four types of communication based on distance: autocrine, juxtacrine, paracrine, and endocrine [174]. Signals are carried between cells through the diffusion and transportation of signal molecules, but they can only be transmitted effectively within a certain range of distances, estimated to be between 10 and 100 cell radii [241,242]. This spatial and distance information is important for understanding cell communication, but it was not accessible from scRNA-seq results until the development of spatial transcriptomics.

Spatial transcriptomics has emerged as a transformative tool for simultaneously recording the gene expression and location of cells. This methodology offers a unique opportunity to characterize the spatial distance tendency of cell–cell communications and therefore to improve the cell–cell communications potential inferred solely by the expression pattern of ligand–receptor pairs. The prediction of cell–cell communications using single-cell RNA sequencing alone is, therefore, insufficient, as spatial distance tendencies and actual cell-type distribution must also be considered. Recent technological advancements have enabled the integration of spatial information with transcriptomic data better to comprehend the spatial organization of cell–cell communication networks. Notable methods that accomplish this include CellPhoneDB v3 [176], Giotto [180], stLearn [185], SpaOTsc [183], SpaTalk [196], and COMMOT [198]. For example, CellPhoneDB v3 focuses specifically on interactions taking place between cell types that exist within the same spatial microenvironment. Notably, in 2D or 3D tissue, cell–cell communication can be measured as a vector with a direction. COMMOT [198] infers such a communication direction by applying optimal transport analysis tools to spatial transcriptomics data, considering the complex interactions between ligands and receptors and constraints of an effective intercellular communication distance. Knowing the location of cells in tissue is crucial for understanding how they communicate and interact with each other. In one study [243], researchers were able to determine the 3D organization of bone marrow cells and identify communication between immune and non-immune cells. Fawkner-Corbeett et al. [244] tested spatial co-localization of identified receptor–ligand pairs by fitting a generalized linear model to expression data.

In spatial transcriptomics and cell–cell communication studies, researchers can draw upon a wealth of tools and methodologies that have emerged throughout the extensive history of spatial statistics research. One noteworthy example involves employing a random effect Gaussian process framework to effectively model the influence of cell–cell interactions on spatial gene expression profiles [245]. With the statistical modeling approach, it would be feasible to explore the impact of the effect of cell–cell communication on the spatial pattern of cellular gene expression.

The inclusion of histological information alongside spatial data is of paramount importance when deciphering cell–cell communication. In the field of cell–cell communication inference, MISTy [246] has embraced a versatile multi-view approach to seamlessly integrate histological and spatial information within diverse contexts. This integration framework, implemented in MISTy, demonstrates the remarkable ability to harmonize a wide range of data modalities, including the rapidly evolving spatial transcriptomics technology for transcriptomics, epigenomics, proteomics, metabolomics [247], and histological imaging, across various spatial scales. Through this comprehensive fusion of distinct data modes, the multi-view integration approach facilitates weighted inference of cell–cell communication, thereby augmenting our comprehension of intricate cellular interactions.

### 5.4. Cell–Cell Communications: Perspective

One of the key challenges in developing algorithms for analyzing cell–cell communication using single-cell RNA sequencing (scRNA-seq) data is the lack of reliable ground truth for the evaluation [174,189,226,227]. Experimental validation of these algorithms is both time consuming and labor intensive. Typically, only the top candidates are selected for experimental testing in one study, and even this candidate list is often filtered based on the researchers’ domain expertise for obvious reasons. Consequently, the evaluation of cell–cell communication using validation experiments provides limited information.

To overcome this limitation, benchmarking and comparative analysis of cell–cell communication methods employ alternative and indirect evaluation approaches. For instance, Liu et al. [226] integrated spatial information to evaluate a cell–cell communication method and discovered that statistical-based approaches exhibited superior performance and produced more consistent results. Similarly, Dimitrov et al. [189] adopted an alternative strategy by utilizing additional data modalities, such as cytokine expression and spatial information, as indirect evaluation measures.

One consistent observation arising from benchmarking and comparative studies is the significant variation, heterogeneity, and limited concurrence observed in the predictions generated by various cell–cell communication methods. This critical finding underscores the challenges inherent in correctly interpreting the results obtained from cell–cell communication predictions and accurately inferring the underlying cellular interactions. It emphasizes the imperative to establish a comprehensive and meticulously designed experimental framework, with a clearly defined purpose aimed at generating high-quality data that can serve as a reliable gold standard for the rigorous evaluation and refinement of these methods.

Cellular communication within multicellular organisms is a highly intricate and dynamic process that operates in both spatial and temporal dimensions. It relies on the exchange of molecular signals, which exhibit diverse temporal profiles ranging from transient to persistent [171]. The temporal dynamics play a critical role in cell–cell communication and, to explore this intricate process, the collection of time series data is invaluable. This approach allows for precise sampling at multiple time points and within appropriate time windows.

The extent of communication encompasses various mechanisms, including autocrine interactions for intracellular communication, paracrine signaling for communication with neighboring cell types, and endocrine signaling for long-range communication through the circulatory system. These communication pathways form a complex network, where individual cells can act as both senders and receivers of signals, with the potential for signal modification during transmission. The impact of signals on cells is influenced by their specific cell type and state. Therefore, capturing the spatial and temporal heterogeneity of cellular states is crucial in order to achieve a comprehensive understanding of cell–cell communication (e.g., [248,249,250]).

Cell–cell communication involves multiple modes of data, such as gene expression, epigenetic modifications, and protein–protein interactions. However, current analytical methods often focus on a single data modality, limiting our comprehensive understanding of the underlying interactions. Hence, there is an urgent need for methodologies that can integrate multiple data modes, enabling a holistic view of cell–cell communication networks. Such approaches are essential to deepen our understanding of the molecular algorithms employed by cells to fulfill their functions effectively [251].

## 6. Summary and Conclusions

In this review, we have focused on scRNA-seq data analysis, emphasizing statistical count modeling, data annotation, data integration, and cell–cell communication.

In count modeling for scRNA-seq data, the choice of appropriate models depends on the presence or absence of unique molecular identifiers (UMIs). For scRNA-seq with UMI counts, zero-inflated models are not necessary for homogeneous cell populations. Commonly used models include the negative binomial or Poisson–lognormal models. On the other hand, for raw read counts without UMI technology, more complex modeling approaches or specific transformations are required to mitigate the impact of PCR amplification biases.

In the context of spatial barcoding techniques, UMIs are commonly employed, and the negative binomial model appears to be a suitable choice. This model allows the parameters to reflect the aggregation nature of multiple cell types within each spatial spot. For high-plex RNA imaging techniques, where the resolution is at the single-cell level and there is no PCR amplification, it is reasonable to consider both Poisson and negative binomial models as adequate choices.

As statistical models for single-cell and spatial transcriptomics are now well-established, future method development could potentially focus on directly working with raw counts instead of relying on normalized values. This approach has the potential to maximize the utilization of information and further advance the field of single-cell and spatial transcriptomics analysis.

Numerous methods have been developed for cell annotation and clustering in single-cell data analysis. Many of these methods rely on reference cell panels or databases, which play a critical role in accurate cell annotation. However, the heterogeneous nature of data generation processes poses a significant challenge for cell annotation and integration. Traditional methods often overlook the hierarchical structure and distinct levels of cell annotation, hindering a comprehensive understanding of cellular diversity. Recognizing this crucial aspect, novel approaches have emerged to address the hierarchical nature of cell annotation and incorporate multiple levels of annotation information.

The availability of high-quality and comprehensive reference databases is pivotal in ensuring robust and precise cell annotation. Overcoming the challenges associated with data heterogeneity and incorporating hierarchical cell annotation structures are important steps toward achieving more accurate and insightful analyses in single-cell research. By embracing these advancements, researchers can effectively navigate the complexities of cellular diversity and unravel the intricate biological processes underlying cell populations.

For data integration, many computational methods have been developed for both single-modal or multimodal mapping, deconvolution, and multimodal fusion. It is important to understand the application setting of each method for efficient application and analysis. These include the assumptions of each method regarding the biological correspondence between data sets, the assumptions regarding batch effects, and what level of output (cell level or gene level) can be provided after integration. As more single-modal and multimodal data accumulates, we believe multimodality based mapping and integrative analysis are going to be important topics, which can help us to gain more insights into understanding individual cell types, their spatial distributions, and the regulatory mechanisms in cell development as well as their responses to external environment.

Considerable efforts have been invested in enhancing the accuracy of inferring cell–cell communication. These efforts involve incorporating downstream gene regulatory network information and integrating spatial transcriptomics data. As the field progresses, the development of multi-omics approaches combined with spatial transcriptomics will provide additional valuable information to further enhance the inference of cell–cell communication and improve our ability to accurately interpret the data.

By employing sound experimental designs, comprehensive characterization of spatial patterns, and temporal dynamics of ligand–receptor interactions, we can significantly advance our understanding of the molecular algorithms employed by cells to process information and make decisions. This integrated approach holds tremendous potential to unravel the intricate mechanisms underlying cell–cell communication and shed light on the complex orchestration of cellular behaviors.

## Figures and Tables

**Table 1:** Platforms and modalities for single-cell technology.

Platform	Modality	Brief Description
Single-Cell Transcriptomics		
scRNA-seq	Gene Expression	These include platforms using UMI counts such as 10x Chromium [12] and raw read counts such as SMART-seq [13]/SMART-seq2 [14].
snRNA-seq	Gene Expression	This method only measures the nuclear transcripts. However, nuclei dissociation may be more applicable when whole-cell dissociation is challenging. In the research we reviewed, scRNA-seq is commonly used as an umbrella term for various single-cell RNA sequencing techniques, including snRNA-seq.
Spatial Transcriptomics		
High-Plex RNA Imaging (HPRI)	Gene Expression with Spatial Information	The resolution is at single-cell or subcellular level. Techniques include in situ sequencing [15], smFISH [16], STARmap [17], MERFISH [18], and seqFISH [19]/seqFISH+ [20].
Spatial Barcoding	Gene Expression with Spatial Information	Align a tissue to a plate with spots encoding the spatial position, and measure transcripts of each spot. Each spot often contains multiple cells. Techniques include spatial transcriptomics [3], 10× Visium (https://www.10xgenomics.com/products/spatial-gene-expression accessed on 25 May 2023), HDST [21], and Slide-seq [22]/Slide-seq2 [23].
Single-Cell Epigenomics		
scATAC-seq	Chromatin Accessibility	Techniques include plate- or array-based methods such as Fluidigm C1 [24], droplet-based methods such as 10x Chromium [25], or split-pooling based methods such as sciATAC-seq [26].
Single-Cell Methylation	DNA Methylation	Techniques include scRRBS [27], snmC-seq2 [28], and scCGI-seq [29].
Single-Cell Multimodality		
10x Multiome (https://www.10xgenomics.com/products/single-cell-multiome-atac-plus-gene-expression accessed on 25 May 2023), SHARE-seq [30]	Gene Expression + Chromatin Accessibility	Simultaneous high-throughput ATAC and RNA expression.
CITE-seq [31]	Gene Expression + Protein Levels	CITE-seq can measure both the transcriptome and cell surface protein.
ASAP-seq [32]	Chromatin Accessibility + Protein Levels	This technique pairs scATAC-seq with detection of the cell surface and intracellular protein markers.

**Table 2:** A survey of databases used for cell annotation.

Database	Data Source	Link
PanglaoDB	scRNA-seq	https://panglaodb.se/markers/PanglaoDB_markers_27_Mar_2020.tsv.gz accessed on 25 May 2023
Hierarchical PanglaoDB	scRNA-seq	https://github.com/JiaLiVUMC/scMRMA/tree/main/data accessed on 25 May 2023
Cellmarker	scRNA-seq +bulk RNA-seq	http://bio-bigdata.hrbmu.edu.cn/CellMarker/download/all_cell_markers.txt accessed on 25 May 2023
CellMatch	scRNA-seq +bulk RNA-seq	https://github.com/ZJUFanLab/scCATCH/raw/master/data/cellmatch.rda accessed on 25 May 2023
SCSig	scRNA-seq	https://data.broadinstitute.org/gsea-msigdb/msigdb/supplemental/scsig/1.0/scsig.all.v1.0.symbols.gmt accessed on 25 May 2023
SaVanT	Microarray	http://newpathways.mcdb.ucla.edu/savant-dev/SaVanT_Signatures_Release01.zip accessed on 25 May 2023
MSigDB	Bulk RNA-seq, Microarray	https://data.broadinstitute.org/gsea-msigdb/msigdb/release/7.2/msigdb_v7.2.xml accessed on 25 May 2023
xCell	Bulk RNA-seq	https://www.ncbi.nlm.nih.gov/pmc/articles/PMC5688663/bin/13059_2017_1349_MOESM3_ESM.xlsx accessed on 25 May 2023
Enrichr ARCHS4 tissues	Bulk RNA-seq	https://maayanlab.cloud/Enrichr/geneSetLibrary?mode=text&libraryName=ARCHS4_Tissues accessed on 25 May 2023
TISSUES 2.0	Bulk RANseq, Microarray	http://tissues.jensenlab.org/ accessed on 25 May 2023

**Table 3:** Scoring methods used for cell annotation.

Method	Description
CTA (Cell-Type Activity)	Sum of the weighted expression
Ucell	Mann–Whitney U statistic
AUCell	Area under the curve
ssGSEA	Rank-based enrichment score
SCSS	Sum of UMI, normalized by library size
GSVA	Kernel density estimation
Singscore	Normalized mean percentile rank
ScType	Cluster summary enrichment score
JASMINE	Approximate mean of gene ranks and the enrichment of the signatures
AddModuleScore (Seurat)	Average expression level
scCATCH	Evidence-based scoring

**Table 4:** Methods used for feature selection.

Method	Description	Reference
DE	Differentially expressed genes	[88]
DD	Differentially distributed genes by Kolmogorov–Smirnov test	
DV	Differentially variable genes by Bartlett’s test	
BD	Bimodally distributed by bimodality index	[94]
DP	Differentially proportioned genes by chi-squared test	
M3Drop	Dropout-based feature selection	[96]
E-test	Entropy-based feature selection	[91]
FEAST	Unsupervised consensus clustering followed by F-test for ranking features	[95]

**Table 5:** Supervised machine learning methods for cell annotation.

Tool	Year	Reference Database	Algorithm	Ref.
SingleR	2019(**)	Built-in celldex (transcriptome of pure cell types)	Spearman	[63]
scmap-cell	2018(**)	Annotated transcriptome	K-nearest neighbor (KNN)	[110]
Garnett	2019(**)	Marker genes	Elastic net regression	[101]
CellAssign	2019(**)	Marker genes	Probabilistic Bayesian model	[108]
scPred	2019(**)	Annotated transcriptome	Support vector machines (SVM)	[98]
singleCellNet	2019(*)	Annotated transcriptome	Random Forest	[99]
CHETAH	2019(*)	Annotated transcriptome	Spearman and confidence	[109]
cellTypist	2022(*)	Annotated transcriptome	L2-regularized logistic regression	[103]
CellBlast	2020	Annotated transcriptome	Neural network-based generative model	[105]
sciBET	2020	Annotated transcriptome	Multinomial-distribution model	[91]
scClassify	2020	Annotated transcriptome	Weighted KNN	[97]
scDeepSort	2021	Annotated transcriptome	Weighted graph neural network	[104]
scBERT	2022	Annotated transcriptome	BERT	[106]
scAnotate	2023	Annotated transcriptome	Random Forest	[100]
TOSICA	2023	Annotated transcriptome	Transformer	[111]

**, citation > 100; *, citation > 50; accessed on 25 May 2023.

**Table 6:** Representative methods for single-cell and spatial transcriptomics integration.

Tool	Input Data Demonstrated	scRNA-seq Data Preprocessing	Methods/Algorithms	Application/Output
Mapping				
scVI	scRNA-seq <-> scRNA-seq	Raw count matrix	Probabilistic modeling, neural networks, variational inference	scRNA-seq cell level and gene level batch correction scRNA-seq mapping
scANVI	scRNA-seq <-> scRNA-seq	Raw count matrix for UMI counts, gene length normalized count for read counts	Probabilistic modeling, neural networks, variational inference	scRNA-seq cell level and gene level batch correction scRNA-seq mapping annotation of single cells from annotated reference cells
MNN/fastMNN	scRNA-seq <-> scRNA-seq	Normalized with the library size, log transformed	Randomized SVD, MNN, weighted average of correction vectors	scRNA-seq cell level and gene level batch correction scRNA-seq mapping
Scanorama	scRNA-seq <-> scRNA-seq	L2-normalized for each cell	Randomized SVD, MNN, weighted average of correction vectors	scRNA-seq cell level and gene level batch correction scRNA-seq mapping
Seurat V3	scRNA-seq <-> scRNA-seq scRNA-seq <-> HPRI scRNA-seq <-> CITE-seq scRNA-seq <-> scATAC-seq	Normalized with the library size, log transformed, gene scaled	CCA, MNN, anchor scoring and weighting	scRNA-seq cell level and gene level batch correction scRNA-seq mapping Multimodal data mapping
Harmony	scRNA-seq <-> scRNA-seq scRNA-seq <-> HPRI	Normalized with the library size, log transformed, gene scaled, PCs from PCA	Maximum batch diversity soft k-means clustering, linear mixture model correction	scRNA-seq cell level batch correction scRNA-seq mapping Multimodal data mapping
LIGER	scRNA-seq <-> scRNA-seq scRNA-seq <-> HPRI scRNA-seq <-> single cell DNA methylation	Normalized with the library size, gene scaled but not centered	Integrative non-negative matrix factorization, shared factor neighborhood clustering	scRNA-seq cell level batch correction scRNA-seq mapping Multimodal data mapping
SpaGE	scRNA-seq <-> HPRI	Normalized with the library size, log transformed, gene scaled	SVD on the cosine similarity matrix of PCs from each modality	Multimodal data mapping
gimVI	scRNA-seq <-> HPRI	Raw count matrix	Probabilistic modeling, neural networks, variational inference	Multimodal data mapping (for gene imputation)
Tangram	scRNA-seq <-> spatial barcoding and HPRI	Normalized with the library size	Direct minimization of a Kullback–Leibler divergence and cosine distances	Multimodal data mapping
Cobolt	Unimodal data sets and multimodal data set	Raw count matrix	Probabilistic modeling, neural networks, variational inference	Multimodal data mapping
MultiVI	Unimodal data sets and multimodal data set	Raw count matrix	Probabilistic modeling, neural networks, variational inference	Multimodal data mapping
Seurat V5	Unimodal data sets and multimodal data sets	Depends on the mapping method in the first mapping step	Dictionary learning, Laplacian eigen-decomposition, sketching	Multimodal data mapping
Deconvolution				
Cell2location	scRNA-seq <-> spatial barcoding data	Raw count matrix	Bayesian negative binomial models, approximate variational inference	Estimate the absolute cell type abundance for each spot of spatial data
RCTD	scRNA-seq <-> spatial barcoding data	Raw count matrix	Poisson–lognormal models	Estimate the proportion of cell types for each spot of spatial data
stereoscope	scRNA-seq <-> spatial barcoding data	Raw count matrix	Negative binomial models	Estimate the proportion of cell types for each spot of spatial data
SpatialDWLS	scRNA-seq <-> spatial barcoding data	Normalized with the library size, log transformed	Enrichment analysis, dampened weighted least squares	Estimate the proportion of cell types for each spot of spatial data
SPOTlight	scRNA-seq <-> spatial barcoding data	Gene scaled	A seeded non-negative matrix factorization (NMF) regression and non-negative least squares	Estimate the proportion of cell types for each spot of spatial data
DestVI	scRNA-seq <-> spatial barcoding data	Raw count matrix	Probabilistic modeling, negative binomial models, neural networks, variational inference	Estimate both the proportion of cell types and the variations within each cell type for each spot of spatial data
Multimodality Fusion				
MOFA+	scNMT-seq, single cell DNA methylation	Normalized with the library size, log transformed	Multi-omics factor analysis	Low-dimension visualization and clustering
totalVI	CITE-seq	Raw count matrix	Joint probabilistic modeling of genes and proteins, neural networks, variational inference	Low-dimension visualization and clustering Multimodality mapping Protein background decoupling, batch correction Protein level imputation Differential expression
Seurat V4	CITE-seq, SHARE-seq, ASAP-seq	Normalized with the library size, log transformed, gene scaled	Supervised PCA, weighted similarity measure from different modalities	Low-dimension visualization and clustering Multimodality mapping

**Table 7:** Cell–cell communication analysis methods (CCC: cell–cell communication; LR: ligand–receptor pair).

Tools	Year	Description	Ref.
CellPhoneDB	2020 (**)	Infer CCC from combined expression of multi-subunit LRs, updated with spatial information integration.	[175,176]
CellChat	2021 (**)	Quantify CCC by a mass action-based model.	[177]
NicheNet	2020 (**)	Infer CCC by incorporating signaling network information.	[178]
CellTalker	2020 (*)	Infer CCC by differentially expressed LRs.	[179]
Giotto	2021 (*)	Identify CCC by incorporating spatial information.	[180]
SingleCellSignalR	2020 (*)	Quantify CCC by regularized ligand–receptor expression product.	[181]
iTALK	2019 (*)	Comparative and longitudinal CCC analysis.	[182]
SpaOTsc	2020 (*)	Infer CCC using an optimal transport model, incorporating downstream expression information.	[183]
CCCExplorer	2015 (*)	Infer CCC by incorporating downstream TF expression.	[184]
stLearn	2020 (*)	Integrating spatial transcriptomics for CCC inference.	[185]
SoptSC	2019 (*)	Score CCC by integrating downstream signaling measurements.	[186]
NATMI	2020	Extensive cell–cell communication network analysis.	[187]
ICELLNET	2021	Integrate LR information for CCC inference.	[188]
LIANA	2022	Infer CCC by a consensus combination of ligand–receptor methods and resources.	[189]
scMLnet	2021	Infer CCC by multilayer network method, which incorporates gene regulatory networks information.	[190]
PyMINEr	2019	Quantify CCC by differentially expressed LRs.	[191]
Connectome	2022	Differential and comparative CCC analysis.	[192]
scTensor	2019	Represent LR data as a tensor and then take decomposition.	[193]
CytoTalk	2021	Use crosstalk score to rank CCC signal.	[194]
Tensor-cell2cell	2022	Using tensor decomposition to characterize context dependent CCC.	[195]
SpaTalk	2023	Integrate spatial information to constrain CCC inference.	[196]
scConnect	2021	Inferring CCC by incorporating interactions in a multi-directional graph.	[197]
COMMOT	2023	Incorporate spatial information and biochemical reaction information to CCC reconstruction.	[198]
Domino	2022	Infer CCC by correlating expression of receptor to expression pattern in gene regulatory network.	[199]
scSeqComm	2022	Identify and quantify CCC through the evidence of ongoing intercellular and intracellular signaling.	[200]
Scriabin	2023	Infer CCC using a binning approach, without cell aggregation or down sampling.	[201]
spaCI	2023	Use adaptive graph model with attention mechanisms to incorporate both spatial locations and gene expression profiles of cells for CCC inference.	[202]
SpatialDM	2022	Spatial association/co-expression of LRs.	[203]
scLR	2022	Examine dysregulated ligand–receptor interactions between two conditions.	[204]
LRLoop	2023	Infer feedback loops in CCC.	[205]
scTenifoldXct	2023	Use manifold alignment for CCC inference, with comparative interaction analysis included.	[206]
DiSiR	2023	Considering interactions that are not listed in reference LR databases.	[207]
Renoir	2023	Infer CCC across a spatial topology and delineate spatial-specific communication niches.	[208]
SPRUCE	2023	Systematically infer common CCC patterns embedded in single-cell RNA-seq data.	[209]
HiVAE	2023	Quantify CCC by transfer entropy with hierarchical variational autoencoder.	[210]
Calligraphy	2023	Utilizes LR modularity to robustly infer the CCC.	[211]
CellCallEXT	2022	Identify LR that alter the expression of downstream genes between two conditions.	[212]

** citation > 500; * citation > 100, accessed on 25 May 2023.

## Data Availability

Not applicable.
